# Microanalysis of β-(Al_x_Ga_1−x_)_2_O_3_ Films Grown by MOCVD

**DOI:** 10.3390/ma19040672

**Published:** 2026-02-10

**Authors:** Mugove Maruzane, Arpit Nandi, Sean Douglas, Lewis Penman, Sai Charan Vanjari, Indraneel Sanyal, Matthew Smith, Robert W. Martin, Martin Kuball, Fabien C. P. Massabuau

**Affiliations:** 1 Department of Physics, Scottish Universities Physics Alliance (SUPA), University of Strathclyde, Glasgow G4 0NG, UK; 2 Center for Device Thermography and Reliability, University of Bristol, Bristol BS8 1TL, UK

**Keywords:** gallium oxide, epitaxy, alloying, bandgap, microanalysis, scanning electron microscopy, cathodoluminescence, atomic force microscopy

## Abstract

A combined microanalysis and optical study of β-(Al_x_Ga_1−x_)_2_O_3_ films grown on sapphire via metalorganic chemical vapour deposition, with thickness 350–1000 nm and Al fraction (x) from 0% to 45%, is presented. Al incorporation in the films showed a linear relation with nominal Al composition calculated from precursor flow rate, and the optical bandgap increased from 4.96 eV to 5.44 eV with a bowing parameter of 1.7 ± 0.5 eV. A high Al fraction led to reduced crystallinity, increased surface roughness, and diminished cathodoluminescence intensity. The topography revealed elongated surface features that evolved with Al content, and luminescence spectra exhibited a blueshift in peak emission attributed to the widening of the bandgap. These findings highlight the trade-off between bandgap tuning and material quality, informing future growth strategies for future electronic and optical devices.

## 1. Introduction

Gallium oxide (Ga_2_O_3_) is an ultra-wide bandgap semiconductor, with much potential for high-power switching and deep UV sensor applications due to its bandgap of ca. 4.5–5.3 eV [1,2,3]. The thermodynamically stable monoclinic β-Ga_2_O_3_ can be grown from the melt or epitaxially, allowing for the growth of high-quality β-Ga_2_O_3_ layers by homoepitaxy on bulk β-Ga_2_O_3_ or by heteroepitaxy on sapphire (α-Al_2_O_3_) [2,4].

In semiconductors, bandgap tuning allows for the development of devices that enhance desired electronic and optical properties. A variety of elements, such as aluminium (Al), indium (In), titanium (Ti), iron (Fe), chromium (Cr), rhodium (Rh), iridium (Ir), tin (Sn), zinc (Zn), and magnesium (Mg) have been successfully incorporated into Ga_2_O_3_ to achieve bandgap tuning of the resulting Ga_2_O_3_-based alloy [5,6,7,8,9,10,11,12,13,14]. β-(Al_x_Ga_1−x_)_2_O_3_ has received particular interest to increase the material bandgap beyond that of β-Ga_2_O_3_. Peelaers et al. reported the theoretical direct bandgap of β-(Al_x_Ga_1−x_)_2_O_3_ to vary between 4.8 eV (for monoclinic β-Ga_2_O_3_) and a maximum of 7.1 eV (for monoclinic ϴ-Al_2_O_3_) with bowing parameters of b = 1.37 eV for a direct bandgap following the quadratic expression E_g (AlxGa1−x)2O3_ = (1 − x)E_g Ga2O3_ + xE_g Al2O3_ − bx(1 − x) [15]. Experimental results have supported these bowing parameter values [16,17,18,19].

(Al_x_Ga_1−x_)_2_O_3_ alloys have been successfully produced using radio frequency magnetron sputtering [20,21], sol-gel spin coating [22,23], Czochralski growth [24,25,26], molecular beam epitaxy (MBE) [16,27,28,29,30,31,32], pulsed laser deposition (PLD) [33,34,35], and metalorganic chemical vapour deposition (MOCVD) [17,18,19,36,37,38,39,40,41,42,43]. Al incorporation into β-(Al_x_Ga_1−x_)_2_O_3_ has been reported across the full range from x = 0 to 100%; however, the quality of the β-(Al_x_Ga_1−x_)_2_O_3_ material varies significantly with the alloy fraction, film thickness and growth method. Peelears et al. performed calculations comparing the enthalpy of formation between the monoclinic and corundum phases, which showed the monoclinic phase is favoured for Al fractions up to x = 71%, beyond which the corundum phase becomes most stable [15]. Czochralski growth of β-(Al_x_Ga_1−x_)_2_O_3_ has been reported to yield single-phase β-(Al_x_Ga_1−x_)_2_O_3_ up to an Al incorporation of 33% and mixed-phase for greater Al fractions [25]. Radiofrequency magnetron sputtering obtained single-phase β-(Al_x_Ga_1−x_)_2_O_3_ films with 400–500 nm thickness and an Al composition x = 42% [21]. Samples made from sputtering had a noticeable decrease in crystalline quality as the Al composition exceeded 70%, with reports describing a lack of crystallinity for samples with an Al composition of 78% and 84% [20,21,22,23]. The highest achieved Al composition using MBE depends on the growth temperature; for example, Oshima et al. reported a maximum concentration of 68% at 800 °C, while Feng et al. obtained a maximum concentration of 35% at 610 °C [16,27]. Literature using PLD describes a decrease in crystallinity with increasing Al composition, with Kranert et al. and Zhang et al. debating the existence of α-phase material past an Al composition of 40% along with the appearance of ϒ–(Al_x_Ga_1−x_)_2_O_3_ [33,34]. MOCVD has become the technique of choice due to its good film quality, control, and the possibility of heterostructures, which enable the epitaxy of β-(Al_x_Ga_1−x_)_2_O_3_ films with thicknesses ranging from 100 to 800 nm [17,18,19,36,37,38,39,40,41,42,43]. Increasing the Al composition in MOCVD thin films of β-(Al_x_Ga_1−x_)_2_O_3_ above x = 27% has been reported to result in the emergence of α and ϒ phases, with a transitional region where β, α and ϒ phases can co-exist for Al contents between x = 27% and 40% [17,18,44]. For Al composition greater than x = 40%, compositional segregation was observed [17,44]. Differences between various growth techniques will likely impact the phase and crystal quality of the β-(Al_x_Ga_1−x_)_2_O_3_ thin films. However, the literature indicates that phase degradation or transitions for β-(Al_x_Ga_1−x_)_2_O_3_ are likely to occur for Al compositions in the 30–40% window [17,18,34,44].

β-(Al_x_Ga_1−x_)_2_O_3_ has been reported to have a broad luminescence that extends from the band-edge region of β-(Al_x_Ga_1−x_)_2_O_3_ to a defect level luminescence of 2.0 eV [19,20,21,26,32,35]. However, the wider bandgap with greater Al composition increases the excitation energy required for electrons to move into the conduction band. Increased excitation energy makes it challenging to perform photoluminescence studies for β-(Al_x_Ga_1−x_)_2_O_3_ with a high Al fraction. This, therefore, brings in cathodoluminescence as a powerful method for studying the luminescence of β-(Al_x_Ga_1−x_)_2_O_3_ [20,21,26,32,35]. In β-Ga_2_O_3_ cathodoluminescence, a high-energy electron beam generates electrons and holes, which are subsequently trapped by the rich library of localised states—including hole polarons, and native and extrinsic donors and acceptors—residing within the bandgap of the semiconductor. Carrier recombination via these states gives rise to a broad luminescence spanning the UV and visible spectral ranges. UV luminescence in the 3.2–3.6 eV range has been ascribed to the recombination of free electrons with self-trapped holes on oxygen sites [19,45,46,47,48], which has also been reported in β-(Al_x_Ga_1−x_)_2_O_3_ [19,20,21,26,32,35]. Blue luminescence in the range of 2.8–3.2 eV for β-Ga_2_O_3_ [45,46,47,49] has also been observed in the same range for β-(Al_x_Ga_1−x_)_2_O_3_ [19,20,21,26,32,35], ascribed to a donor–acceptor pair transitions involving oxygen vacancies (V_O_), gallium vacancies (V_Ga_) or divacancy complexes of gallium and oxygen (V_Ga_-V_O_) [19,20,21,26,35,45,46,47,49]. Green luminescence in the range of 2.0–2.7 eV has been attributed to donor–acceptor pair transitions involving oxygen interstitials (O_i_) and clusters of oxygen vacancies, which form complexes in β-Ga_2_O_3_ [49] and β-(Al_x_Ga_1−x_)_2_O_3_ [20,21,32]. Yuan et al. reported a near band-edge deep UV luminescence peak at 5.7 eV on a 120 nm thick β-(Al_0.05_Ga_0.95_)_2_O_3_ film grown by PLD [35]. The intensity of the luminescence of β-(Al_x_Ga_1−x_)_2_O_3_ has also been reported to reduce with increasing Al composition, indicating that the addition of Al results in an increase in non-radiative transitions [19,20,32].

Studies on β-(Al_x_Ga_1−x_)_2_O_3_ films with a thickness above 350 nm remain limited, and their properties are not yet fully understood. Gaining insight into these thick films is essential for optimising MOCVD growth techniques for device applications. In this work, we present a microanalysis of 350–1000 nm thick β-(Al_x_Ga_1−x_)_2_O_3_ films grown on *c*-plane sapphire by MOCVD. We use a combination of wavelength dispersive X-ray spectroscopy (WDX), secondary electron microscopy (SEM), atomic force microscopy (AFM), X-ray diffraction (XRD), and cathodoluminescence (CL) spectroscopy to provide insights into the effect of Al composition on surface topography, defect formation, crystallinity, and phase evolution of the films.

## 2. Experimental Methods

Films of non-intentionally doped β-(Al_x_Ga_1−x_)_2_O_3_ with thicknesses ranging from 350 to 1000 nm were grown using an Agnitron Agilis 100 MOCVD system (Agnitron, Chanhassen, MN, USA) on *c*-plane sapphire substrates. Triethylgallium (TEGa) and triethylaluminium (TEAl) were used as Ga and Al precursors, respectively, and O_2_ and Ar were used as carrier gases. All the β-(Al_x_Ga_1−x_)_2_O_3_ films were grown at 800 °C, while β-Ga_2_O_3_ was grown at 840 °C. All the films were grown with the same O_2_ flow rate of 800 sccm. Variations in the Al fraction of the films were obtained by modulating the metal precursor molar flows, later referred to as x_growth_ = TEAl/(TEGa + TEAl). We aimed to keep the Al composition below x = 40% due to the reported β-(Al_x_Ga_1−x_)_2_O_3_ phase separation discussed previously [17,18,34,44]. Table 1 summarises the measured thickness and expected Al composition calculated from the precursor flow rate.

XRD was conducted in a Philips X’pert system (Malvern Panalytical, Malvern, UK) with a Cu Kα1 radiation source to monitor the crystallinity and phase of the films. AFM in a Bruker Edge system was employed to obtain the surface morphology of the samples. The thickness of the films was determined by reflectometry and cross-checked by cross-sectional SEM. Transmittance data were collected using a Shimadzu UV-2600 UV–vis spectrophotometer equipped with an ISR-2600Plus integrating sphere (Shimadzu, Kyoto, Japan). The bandgap of all the samples was determined using the absorption edge from a linear interpolation of the α^2^ vs. hν plot, where α is the absorption coefficient, and hν is the energy, as is customary for direct bandgap semiconductors [50]. This model is widely used in the literature and is supported by theoretical predictions showing a small difference between direct and indirect bandgap energies over the entire compositional range [15].

Microanalysis was conducted using SEM, WDX, and CL in a JEOL JXA-8530F field emission electron probe micro-analyser (EPMA) (JEOL, Tokyo, Japan). WDX brings advantages of high spectral resolution and high sensitivity, and also allows CL spectra to be collected at the same time and from exactly the same excited spot as the WDX by using the EPMA’s optical microscope, which is coaxial with the electron beam [51]. Surface topography-sensitive secondary electron images were acquired using acceleration voltages of 5–7 kV. Elemental compositions were measured by WDX using an acceleration voltage of 6 kV and a beam current of 10 nA with a probe diameter of 20 μm, averaging over 9 randomly chosen points on an area of roughly 1–2 mm^2^. Monte Carlo simulations indicated an interaction depth of ca. 180 nm for a 6 kV beam impinging on an (Al_x_Ga_1−x_)_2_O_3_ film (for x = 15%, close to the average x_growth_ value) [52], confirming that the substrate will not interfere with the measurements. The elemental composition was quantified by WDX using α-Al_2_O_3_ (sapphire) for Al standard, and MOCVD β-Ga_2_O_3_ on sapphire for Ga and O standards. The sapphire standard was C-coated due to significant charging, introducing uncertainties when uncoated. Luminescence spectra were acquired at room temperature using an acceleration voltage of 8 kV and a beam current of 20 nA—leading to an interaction depth of ca. 270 nm [52]—which was adequate to obtain a detectable CL signal without inducing significant sample charging. The CL signal was spectrally resolved using a 125 mm focal length spectrometer with a 400 lines/mm diffraction grating blazed at 500 nm. The resulting CL spectra were corrected for system response using the transition radiation of pure Al [53].

## 3. Results and Discussion

**Figure 1:**
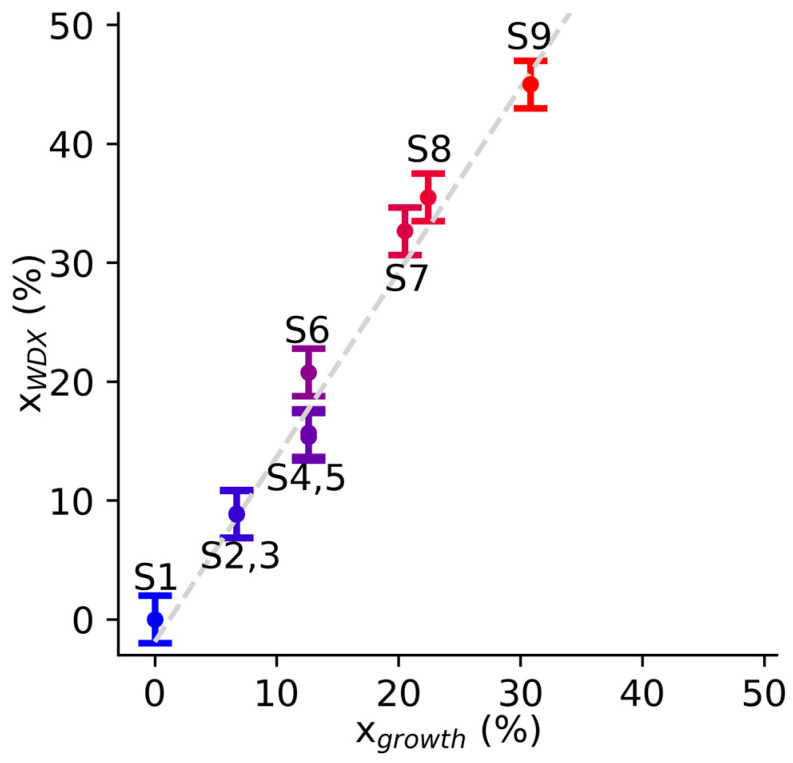
Comparative plot of Al content estimated through the precursor flow rate (x_growth_) vs. Al content measured by WDX (x_WDX_).

Figure 1 shows the relation between the Al fraction expected from growth (x_growth_), based on the TEAl flow rate, and the Al fraction incorporated in the film measured by WDX (x_WDX_). When performing WDX measurements, the error bar was calculated by adding the random error, obtained from the standard deviation of the 9 measurement points (0.5–1%, rounded up to 1%), to the WDX systematic error of 1%—the error bars being dominated by instrumental uncertainty suggests good compositional homogeneity across the sample surface. The graph shows a linear relation (R^2^ = 0.977) between the flow rate Al fraction and the WDX Al fraction. We observe a super-linear trend (x_WDX_ > x_growth_), which we attribute to unequal cation incorporation efficiencies for the growth conditions used here—previous reports have commented on Al incorporation dependence on growth conditions and substrate orientation [7,54]. We see minimal to negligible impact of thickness on the measured composition for most samples, exemplified by samples S2 and S3, which have the same composition (x_WDX_ = 8.8%) but different thicknesses (468 nm and 765 nm, respectively), as well as samples S4 and S5 (x_WDX_ = 15.4% but thicknesses 392 nm and 788 nm, respectively). This suggests that the film composition is relatively uniform across its thickness. S6 is an exception, having the same growth conditions as samples S4 and S5 but different x_WDX_ = 20.8% and thickness of 1000 nm. Compositional inhomogeneities can arise from structural defects or from a pulling effect (compositional gradient in the film caused by lattice mismatch with the substrate) during growth [55]. A more detailed analysis would, however, require cross-sectional nanoscale analysis, which was not possible here due to the samples charging quickly under the electron beam.

**Figure 2:**
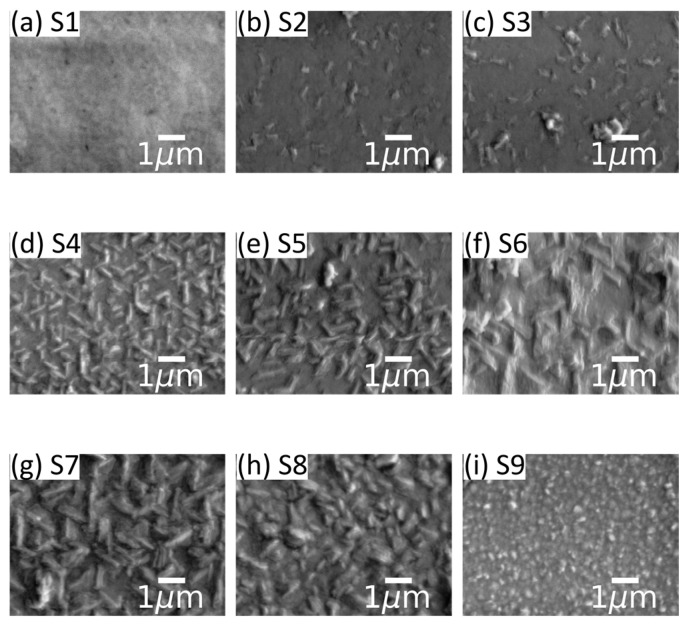
SEM micrographs of the samples.

Figure 2 shows SEM micrographs revealing the topography of the sample set. The topography of S1 (x_WDX_ = 0%) shows a very smooth surface with no visible features. As the Al fraction increases to x_WDX_ = 8.8% for S2 (thickness 468 nm) and S3 (thickness 765 nm), we observe the appearance of small features protruding from the film. These elongated features with a length of ca. 0.2 μm are sparsely distributed across the film surface with estimated densities of ca. 0.8 × 10^8^ cm^−2^ and 1.0 × 10^8^ cm^−2^ for S2 and S3, respectively. Further increasing the Al fraction to x_WDX_ = 15.4% (S4, thickness 392 nm) and x_WDX_ = 15.7% (S5, thickness 788 nm), the films exhibit more and bigger elongated features that protrude from the film. These features, with a length of ca. 1 μm and a width of ca. 50–100 nm, occur at densities of ca. 3.4 × 10^8^ cm^−2^ and 1.8 × 10^8^ cm^−2^, respectively, and are oriented at 60° or 120° to each other. With a further increase in the Al fraction to x_WDX_ = 20.8% (S6, thickness 1000 nm) and x_WDX_ = 32.7% (S7, thickness 1000 nm), we observe that these elongated features increase in length to ca. 1.5 μm and in width to 50–150 nm, with estimated densities of ca. 1.0 × 10^8^ cm^−2^ and 1.2 × 10^8^ cm^−2^, respectively. Bhuiyan et al. observed similar features in AFM data and reported that the features propagate along the [010] and [100] directions [37,39]. Based on the angular nature of the features we observe, we can speculate that the features we observe in Figure 2d–g are similar. When the Al fraction reaches x_WDX_ = 35.5% (S8, thickness 630 nm), the topography exhibits a mixture of smaller elongated features with small circular features, totalling an estimated density of ca. 1.9 × 10^8^ cm^−2^. The elongated features exhibit a broader spread in dimensions, with a length varying from 0.1 to 1 μm. Lastly, the topography of S9 (x_WDX_ = 45%, thickness 724 nm) shows only small circular features with sizes of less than 200 nm and an estimated density of ca. 6.1 × 10^8^ cm^−2^. Significant sample charging under the electron beam prevented high spatial resolution WDX or EDX compositional mapping of the elongated surface features.

**Figure 3:**
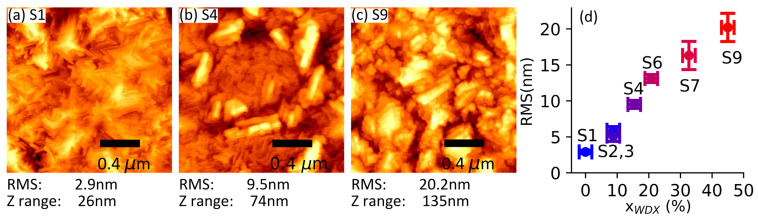
AFM image for samples S1 (**a**), S4 (**b**), and S9 (**c**), and a plot showing the evolution of the RMS roughness vs. Al composition (**d**).

The topography of the samples was investigated at a 1 μm × 1 μm scale using AFM, as shown in Figure 3. The AFM micrographs are consistent with the wider field-of-view micrographs from SEM (Figure 2)—i.e., with elongated features appearing and increasing in size with the Al fraction—shown in Figure 3a–c for samples S1, S4 and S9. We can see that the surface of the pure Ga_2_O_3_ sample S1 shows a very smooth surface made up of small features that are at an angle of roughly 60° to each other, while sample S4 (x_WDX_ = 15.4%) exhibits several elongated features, with a length of ca. 0.3 μm and width of ca. 0.1 μm, oriented at 60° to each other, consistent with the SEM images in Figure 2. These run along the family of <010> directions based on observations made by Bhuiyan et al. [37]. Figure 3d plots the RMS roughness against Al composition, showing a monotonous increase in roughness with increasing Al fraction.

**Figure 4:**
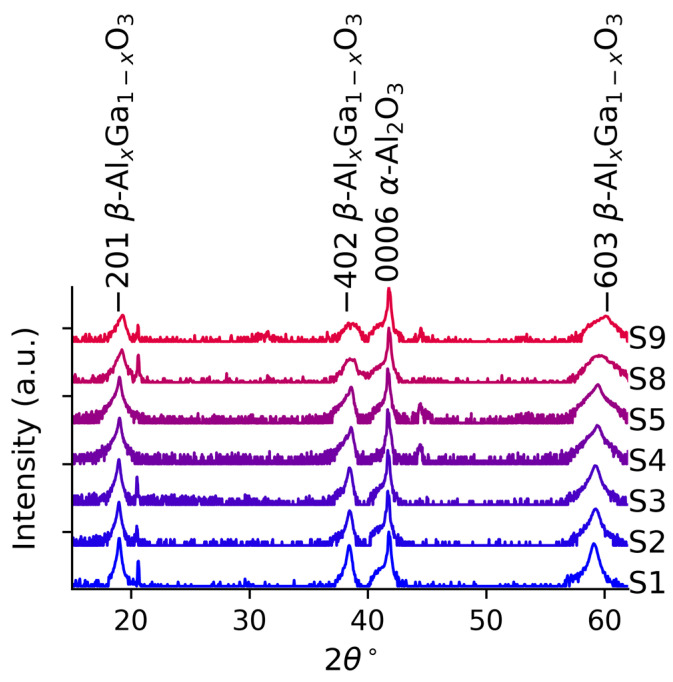
ω-2θ XRD diffractogram of the samples.

Figure 4 shows the symmetric ω-2θ XRD scans of the samples. All the diffractograms contain three peaks related to the β-(Al_x_Ga_1−x_)_2_O_3_ film, at approximately 18°, 38°, and 59° corresponding to the $\bar{2}01$, $\bar{4}02$, and $\bar{6}03$ reflections [56,57,58], confirming that all the films are β-phase and dominantly $\left(\bar{2}01\right)$ oriented. The peak near 41° corresponds to the 0006 reflection of the sapphire substrate. For x_WDX_ > 15%, we observe the emergence of weak reflections near 45°, which could correspond to the 004 reflections of ϒ-(Al_x_Ga_1-x_)_2_O_3_ [59], and for S9 we also observe a low-intensity reflection near 32°, which corresponds to the 220 reflections of ϒ-(Al_x_Ga_1-x_)_2_O_3_ [59,60], indicative that this film is not entirely phase pure, as expected for such high Al content, and in agreement previous literature on homoepitaxial thin films [17]. We observe that the peaks gradually weaken and broaden as the Al fraction increases, which can partially be explained by the lower atomic scattering factor of Al compared to Ga [61] but is mainly indicative of a gradually decreasing crystalline quality of the films [61,62], and corroborates the increase in surface roughness observed by AFM and SEM.

**Figure 5:**
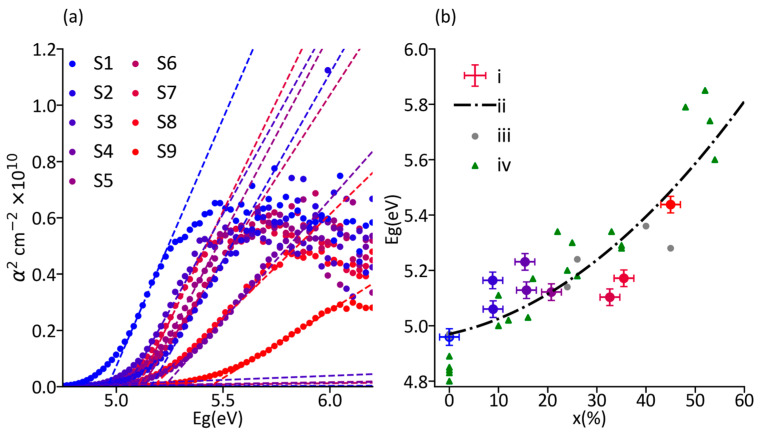
Plots of (**a**) α^2^ vs. hν, and (**b**) x_WDX_ vs. bandgap for each of the (Al_x_Ga_1−x_)_2_O_3_ samples, (i) bandgap values from Samples S1–S9 with colours following those in the legend in (**a**), (ii) line of best fit for the relation between bandgap and Al composition, (iii) additional samples from our group, and (iv) datapoints from refs [16,19,37,39].

The optical bandgap was measured using UV–vis spectrophotometry and obtained from the intersection of the linear fits of the squared absorption coefficient (α^2^) edge with the baseline. S1 exhibits a bandgap of 4.96 eV, in excellent agreement with the reported bandgap of β-Ga_2_O_3_ [1,2,3,47], and we observe a clear shift of the absorption edge to greater energies as the Al fraction increases (Figure 5a). We also observe that the edge becomes less steep as the Al composition increases, which indicates a greater disorder and lower crystalline quality of the films [17,18,34], in line with our XRD analysis and increasing density and size of topographical features in AFM and SEM. Figure 5b compiles the results from bandgap measurement with Al quantification by WDX (x_WDX_) and compares against the literature [15,16,17,34,37,39]. Small differences in bandgap energies from other sources may originate from a different relaxation state of the films and the methods used to determine the bandgap and film composition. For example, Bhuiyan et al. analysed 65 nm thick samples using X-ray photoelectron spectroscopy to obtain composition and bandgap [39,63], which will naturally lead to minor discrepancies with our data. We observe an impact of the film thickness in the bandgap value, where for identical Al composition, the thicker films exhibit slightly lower bandgap—for example, samples S2 and S3 have a slight bandgap variation of ca. 0.1 eV, which can be attributed to variations in the strain state of the film [64]. Figure 5b shows the quadratic line of best fit across samples S1–S9, affording a bowing parameter of b = 1.7 ± 0.5 eV in agreement with other reports [15,37,39]. To minimise the number of fitting parameters, the bandgap energy of β-Ga_2_O_3_ was fixed at 4.96 eV, a value in which we have high confidence due to its excellent match with the literature, while the bandgap of monoclinic Al_2_O_3_ was allowed to vary within the range reported in the literature (7.0–7.5 eV), yielding a value of 7.05 eV [15,16,31,39].

**Figure 6:**
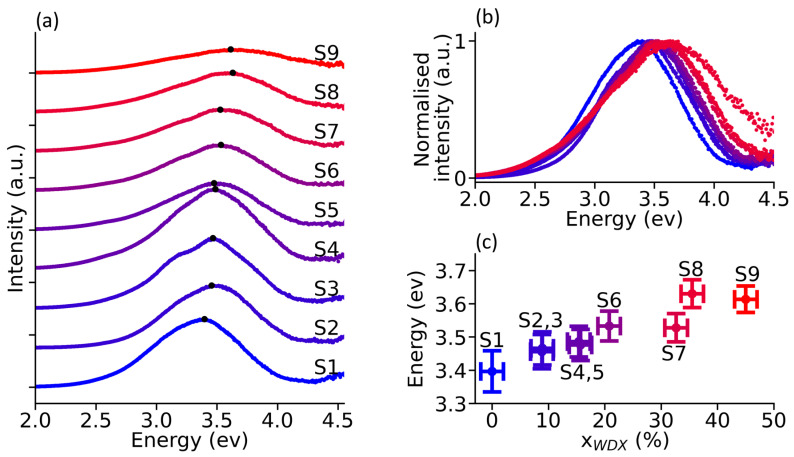
(**a**) CL spectra taken at 300 K, (**b**) normalised CL spectra, and (**c**) evolution of the peak intensity vs. Al composition.

Figure 6a shows a plot of the CL spectra recorded for each sample. The figure shows that the luminescence of β-(Al_x_Ga_1−x_)_2_O_3_ has similar broad luminescence to that observed for β-Ga_2_O_3_ [19,20,21,26,32,35]. The luminescence spectra for samples S1–S4 exhibit similar intensity, but the intensity is nearly halved for samples S5–S8, and reduced by a factor of 4 for S9. This gradual decrease in luminescence with Al composition is consistent with the decrease in crystalline quality we observed by XRD, the occurrence of elongated features observed by AFM, and the shallower absorption edge observed by UV–vis spectrophotometry. Figure 6b overlays normalised spectra and shows that the main luminescence variations are mainly located on the high-energy side of the spectra, with comparatively little variation on the low-energy side. We observe a decrease in the CL intensity and a blueshift with increasing Al composition, and Figure 6c illustrates the blueshift of the peak intensity vs. x_WDX_. The peak intensity blueshifts with increasing Al composition, reaching a maximum of 0.24 eV between the two extreme samples (S1 and S9). Samples with the same composition but differing thickness did not have any identifiable blueshifts. According to theoretical calculations, the luminescence energy from the recombination of self-trapped holes with free electrons should be unaffected by the widening of the bandgap due to increasing Al composition, and only the donor–acceptor pair transitions should be affected [32]. This is, however, nuanced by our results, as well as other experimental reports that show self-trapped hole luminescence blueshifting by ca. 0.1 eV for Al compositions ranging between x = 0% and 40–45% [15,20,32]. The blueshift can also be a result of the emergence of a separate peak related to the increasing Al composition in the samples, since pure Al_2_O_3_ has been reported to exhibit an F-centre at 3.8 eV [65]. The scarcity of detailed defect studies on the evolution of defect state energies as a function of Al composition prevents us from determining whether the observed luminescence shift arises from an energy shift of individual luminescence bands or from a change in their relative intensities. More research would be required to gain a better understanding of this.

Table 2 summarises all the key information from the data discussed above. The last column in Table 2 shows that Al composition in β-(Al_x_Ga_1−x_)_2_O_3_ increases the material’s bandgap, as shown in the transmittance data. However, due to the instability of the monoclinic phase Al_2_O_3_, increasing Al composition results in a degradation of the structure, exemplified by a lower XRD intensity. Lower crystalline quality upon increasing Al fraction is also visible through the shallower absorption edge in Figure 5 and reduced CL intensity in Figure 6. Another sign of reduced crystal quality upon increasing Al composition is the increasing RMS roughness through the appearance of surface features with increased density and size, as shown in Figure 2 and Figure 3. The decreased crystal quality, observed through the degradation of the films’ structural and optical properties, most likely suggests the generation of non-radiative recombination centres in the films, which will be important to alleviate for future deep-UV optoelectronic and power electronic devices.

## 4. Conclusions

β-(Al_x_Ga_1−x_)_2_O_3_ films with Al composition up to x = 45% and thicknesses up to 350–1000 nm were grown using MOCVD on *c*-plane sapphire and characterised using SEM, AFM, XRD, WDX, UV–vis and CL. We observed that Al incorporation in the films showed a linear relation with precursor flow rate, and the optical bandgap increased from 4.96 eV to 5.44 eV, with a bowing parameter of 1.7 ± 0.5 eV. Topographical analysis shows that the roughness of the samples increases with Al composition, with the appearance of elongated features. CL spectra exhibited a decrease in intensity with Al fraction, as well as a blueshift in peak emission attributed to the widening of the bandgap. These findings highlight the trade-off between bandgap tuning and material quality, informing future growth strategies for future electronic and optical devices.

## Figures and Tables

**Table 1:** Sample thickness measured by reflectometry and nominal Al composition based on the growth parameters.

Sample	S1	S2	S3	S4	S5	S6	S7	S8	S9
Thickness (nm)	590	470	760	390	790	1000	1000	630	720
x_growth_ (%)	0	6.7	6.7	12.6	12.6	12.6	20.5	22.4	30.8

**Table 2:** Summary of the key characterisation results for all the samples.

Sample Name	Thickness (nm)	x_growth_ (%)	σ_RMS_ (nm)	x_WDX_ (%)	E_g_ (eV)
S1	590	0	3 ± 1	0	4.96 ± 0.03
S2	470	6.7	6 ± 3	8.8 ± 2	5.16 ± 0.03
S3	770	6.7	5 ± 3	8.8 ± 2	5.05 ± 0.03
S4	390	12.6	9 ± 3	15.4 ± 2	5.23 ± 0.03
S5	790	12.6		15.7 ± 2	5.13 ± 0.03
S6	1000	12.6	13 ± 5	20.8 ± 2	5.12 ± 0.03
S7	1000	20.5	16 ± 6	32.7 ± 2	5.10 ± 0.03
S8	630	22.4		35.5 ± 2	5.17 ± 0.03
S9	720	30.8	20 ± 4	45.0 ± 2	5.44 ± 0.03

## Data Availability

The data presented in this study are openly available in the University of Strathclyde KnowledgeBase at https://doi.org/10.15129/f1a08df5-cdbc-40ae-a01c-1c04fff5b374.
